# Manganese-Induced Neurotoxicity through Impairment of Cross-Talk Pathways in Human Neuroblastoma Cell Line *SH-SY5Y* Differentiated with Retinoic Acid

**DOI:** 10.3390/toxics9120348

**Published:** 2021-12-09

**Authors:** Raúl Bonne Hernández, Nadja C. de Souza-Pinto, Jos Kleinjans, Marcel van Herwijnen, Jolanda Piepers, Houman Moteshareie, Daniel Burnside, Ashkan Golshani

**Affiliations:** 1 Laboratory of Bioinorganic and Environmental Toxicology—LABITA, Department of Chemistry, Federal University of São Paulo, Rua Prof. Artur Riedel, 275, Diadema 09972-270, SP, Brazil; 2 Department of Biology, Carleton University, 209 Nesbitt Biology Building, 1125 Colonel by Drive, Ottawa, ON K1S 5B6, Canada; houman_mot@yahoo.com (H.M.); daniel.j.burnside@gmail.com (D.B.); ashkan_golshani@carleton.ca (A.G.); 3 Departamento de Bioquímica, Instituto de Química, Universidade de São Paulo (USP), Av. Prof. Lineu Prestes, 748, Butantã, São Paulo 05508-900, SP, Brazil; nadja@iq.usp.br; 4 Department of Toxicogenomics, Maastricht University, Universiteitssingel 50, Room 4.112 UNS 50, 6229 ER Maastricht, The Netherlands; j.kleinjans@maastrichtuniversity.nl (J.K.); m.vanherwijnen@maastrichtuniversity.nl (M.v.H.); j.piepers@maastrichtuniversity.nl (J.P.)

**Keywords:** manganese speciation, *SH-SY5Y*, neurotoxicity, neurodegeneration, protein metabolism

## Abstract

Manganese (Mn) is an important element; yet acute and/or chronic exposure to this metal has been linked to neurotoxicity and neurodegenerative illnesses such as Parkinson’s disease and others via an unknown mechanism. To better understand it, we exposed a human neuroblastoma cell model (*SH-SY5Y*) to two Mn chemical species, MnCl_2_ and Citrate of Mn(II) (0–2000 µM), followed by a cell viability assay, transcriptomics, and bioinformatics. Even though these cells have been chemically and genetically modified, which may limit the significance of our findings, we discovered that by using RA-differentiated cells instead of undifferentiated *SH-SY5Y* cell line, both chemical species induce a similar toxicity, potentially governed by disruption of protein metabolism, with some differences. The MnCl_2_ altered amino acid metabolism, which affects RNA metabolism and protein synthesis. Citrate of Mn(II), however, inhibited the E3 ubiquitin ligases–target protein degradation pathway, which can lead to the buildup of damaged/unfolded proteins, consistent with histone modification. Finally, we discovered that Mn(II)-induced cytotoxicity in RA-*SH-SY5Y* cells shared 84 percent of the pathways involved in neurodegenerative diseases.

## 1. Introduction

Manganese (Mn) is the twelfth most abundant element in the crust. Environmentally, it ranges from 1–200 g/L in fresh water to 410–6700 mg/kg (dry weight) in sediments [1]. Mn is a trace mineral that is found in low concentrations in legumes, pineapples, beans, nuts, tea, and cereals [2,3]. Mn is also a key cofactor for enzymes such as glutamine synthetase, pyruvate decarboxylase, serine/threonine protein phosphatase I, Mn-superoxide dismutase, and arginase [4]. Consequently, it is an essential element to maintain normal physiological development including the metabolism of lipid, protein, and carbohydrate; blood sugar regulation; bone formation; immunological response; reproduction; neurotransmitter synthesis and metabolism, as well as neuronal and glial function [3].

Local levels of Mn in the environment can be dramatically increased due to natural and human causes [5,6,7,8,9,10]. Additionally, it can be found as Mn(II), Mn(III), and Mn(IV) in aquatic systems owing to oxi-reductive processes [1,11,12,13,14,15]; these chemical species are environmentally and toxicologically relevant [10,16,17]. Occupational exposure [17] and consumption of contaminated well water [7] represent the most relevant means by which humans are exposed to Mn, with high risks for health [7,17,18,19,20], consistent with increasing evidence of developmental neurotoxicity due to oral parenteral nutrition [7,21].

Mn neurotoxicity, which is characterized by motor and sensory problems, known as manganism, as well as neuropsychiatric and cognitive impairments [3], is the most serious adverse consequence of this metal. Hypertonia with cogwheel stiffness, bradykinesia, “cock-gait”, fast postural tremor, and a tendency to stumble when walking backwards are all symptoms of parkinsonism [3]. These symptoms have been linked to an excess of Mn in the basal ganglia, especially the globus pallidus, subthalamic nucleus, substantia nigra, and striatum, which are involved in motor control and nonmotor functions [3,4]. However, additional brain areas, such as the cerebellum, red nucleus, pons, cortex, thalamus, and anterior horn of the spinal cord, may be altered by Mn exposure [14]. These cellular features, including Mn-induced mitochondrial dysfunction, inflammation, autophagy, overexpression of α-synuclein (αSyn) in vitro, and their aggregation in vivo in neurons and glial cells, have been linked to Parkinson’s disease (PD) [4]. These traits distinguish parkinsonism, which is characterized by the lack of Lewy bodies (another hallmark of PD). Surprisingly, dopaminergic neurons of the substantia nigra pars compacta are particularly destroyed following chronic or acute Mn exposure [3].

Furthermore, Mn toxicity has been connected to Huntington’s disease (HD), since cultured striatal cells surprisingly reduced the vulnerability of mutant expressing cells ST*Hdh*Q111/Q111 [22]. Furthermore, pre-manifest YAC128 transgenic mice, another model of HD, exposed to MnCl_2_ had a decreased response to transcriptional and protein alterations, whereas manifest YAC128 animals had a suppressed metabolic response, despite equivalent elevations in whole striatal Mn [23]. Mn has also been linked to Amyotrophic Lateral Sclerosis (ALS), since certain ALS patients have T1-weighted hyperintensity during MRI, a neuroradiological signal associated with Mn overload, as well as an increase in MnSOD levels in motor neurons and genetic variations of two melastatins, TRPM2 and 7. Whereas early study in macaques has suggested that chronic Mn treatment induces upregulation of amyloidlike protein 1 and diffuse amyloid-β plaques in the frontal cortex, perhaps implying a relationship between advanced-stage manganism and Alzheimer’s disease (AD) [4]. In addition, Mn exposure in dogs enhanced the expression of nuclear neuronal NF-B and iNOS, as well as changed blood–brain barrier (BBB) function, diffuse Aβ plaques, neurofibrillary tangles, and alteration of Mn-dependent antioxidant enzyme, as reported in nonhuman primates and humans [24]. All these alterations can be influenced by chemical fractionation and speciation, developmental stage [14,16,25], and cell type [14].

The cytotoxicity of chemical compounds of Mn and their mechanisms has been supported by several works in silico, in vitro, and in vivo. In this manner, both undifferentiated and differentiated human neuroblastoma cell models (*SH-SY5Y*) were employed to investigate the role of Mn in neurotoxicity [26,27,28]. However, some issues must be considered. For example, undifferentiated *SH-SY5Y* cells can have fluctuations in the cell cycle and are considered immature catecholaminergic neurons [29]. While retinoic acid (RA) synchronizes the cell cycle and generates a modest rate of proliferation, RA differentiates cell morphologically close to primary neurons and increases electrical excitability of the plasma membrane [29], which could leave axons more susceptible for chemical injury [30]. Furthermore, NoRA *SH-SY5Y* cells are disabled in ATP production [31] while RA induces survival of *SH-SY5Y* cells. Consequently, RA-differentiated cells are more resilient to toxins [29]. Altogether, the in vitro cell model should mimic the phenotypes and be sensitive to cellular alterations commonly verified in vivo and specifically in humans [30,31]. It this respect, it has been shown that RA differentiates human neuroblastoma *SH-SY5Y* cells, which generate a largely mature dopaminergic (DAergic)-like neurotransmitter phenotype found in vivo as well as other neurotransmitters in lower expression, such as noradrenaline, acetylcholine, glutamate, serotonin, and histamine [31]. This allows the study of PD, ALS, AD, and HD [31,32,33] together with the relationship between these illnesses and Mn-induced neurotoxicity [26,27,28], potentially governed by disturbance of protein synthesis [25,34,35].

Protein synthesis is an energy-intensive process that is highly controlled and tightly linked to other cellular activities such as the cell cycle and metabolic pathways [36,37]. Previous research with NoRA *SH-SY5Y* cells revealed that MnCl_2_ causes endoplasmic reticulum (ER) stress [38,39], accompanied by autophagy [38], and accumulation of parkin protein and its redistribution to aggregated Golgi complex [39]. Two other independent transcriptomics studies verified that MnCl_2_ induces cytotoxicity in *SH-SY5Y* cells by promoting mitophagy through BNIP3-mediated oxidative stress [40] and/or upregulation of apoptotic pathways, neuronal differentiation, and synaptic transmission [26]. However, they have not identified alteration in the ER–Golgi system, involved in protein metabolism before energy–mitochondrial dysfunction and cell death [28].

However, the research described above has not contemplated the significance of chemical speciation. It is well known that the MnCl_2_ (aqua-complex of Mn or Mn-free) can cross the brain–blood barrier easier than Citrate of Mn(II) or other species of Mn(II) or Mn(III) [41]. In cerebellar granule neurons, the MnCl_2_ was more bioconcentrated than Citrate of Mn(II), although both species displayed similar cytotoxicity, associated with energy–mitochondrial impairment [14]. However, this energetic dysfunction reduced the influx of the two species of Mn in rat brain [42]. In zebrafish embryos, after exposure to species of Mn(II) or Mn(III), the Mn appeared in fluids mainly as Mn-free, followed by Citrate of Mn(II), a species that induced more bioaccumulation of Mn and gene overexpression than MnCl_2_, but both species perturbed the calcium homeostasis and protein metabolism [16,35].

Finally, considering the issues stated above, we hypothesized that impairment of pathways linked to protein biosynthesis drives Mn-induced neurotoxicity and potentially neurodegeneration, which can be affected by chemical speciation. Thus, we aimed to develop a toxicogenomics study in the RA-differentiated *SH-SY5Y*-DAergic cell model, exposed to MnCl_2_ and Citrate of Mn(II). Using system biology approaches, we provided additional evidence that connects Mn-induced impairment of protein metabolism to Mn-neurotoxicity to neurodegenerative disorders (AD, ALS, HD, and PD).

## 2. Materials and Methods

### 2.1. Preparation of Manganese Species

Compounds of manganese (MnCl_2_ and Citrate of Mn(II) or Mn(II)Cit, 10 mM each) were prepared and characterized according to previous works of our group [14,16] and stored at 4 °C. For these experiments we used manganese(II) chloride tetrahydrate, MnCl_2_·4H_2_O (99.99% trace metals basis, Merck, São Paulo, SP, Brazil) and sodium citrate tribasic dihydrate (Cit; HOC(COONa)(CH_2_COONa)_2_·2H_2_O) (99.99% trace metals basis, Merck, São Paulo, SP, Brazil). The work solution was prepared the same day of cell exposure by diluting an aliquot to reach the desired final concentration, according to each experiment.

### 2.2. Human Neuroblastoma SH-SY5Y Cell Line Experimental Setup

Human neuroblastoma *SH-SY5Y* cells, a third generation subclone of *SK-SN-SH* cells, are a brain-derived catecholaminergic neuroblastoma cell line. *SH-SY5Y* cells differentiate into neuronlike cells and cease proliferating [43]. *SH-SY5Y* cells were grown in Dulbecco′s Modified Eagle′s Medium/Nutrient Mixture F-12 Ham (DMEM/F12 nutritional mixture (1:1), Merck, São Paulo, SP, Brazil) supplemented with 10% of Fetal Bovine Serum (FBS, Merck, São Paulo, SP, Brazil) and penicillin/streptomycin (50 IU/mL, Merck, São Paulo, SP, Brazil) in a humidified environment of 5% CO_2_ and 95% air at 37 °C. The medium was changed every 4–5 days. Serum was lowered to 2% FBS for all experimental conditions. Cells were differentiated for 7 days in the presence of 10 nM of trans-Retinoic Acid (trans-RA, Merck, São Paulo, SP, Brazil). Cultures were inspected under a microscope prior to treatment to determine differentiation. When 80 percent or more of the cells in a culture showed neurite outgrowth extensions >2–3 times longer than the cell’s body diameter, the culture was declared differentiated. In the presence or absence of the Mn species, the cells were seeded at a density of 5 × 10^4^ cells per 100 mL in a 96-well plate format for cell viability assay and 6-well plates for array experiment.

### 2.3. Cell Viability Assay

Cell viability was determined in quintuplicate from three separate cell cultures using the MTT test, which measures the reduction of 3-[4,5-dimethythiazol-2-il]-2,5-diphenyl-tetrazolium bromide (MTT, Merck, São Paulo, SP, Brazil) by live cells. We conducted the assay according to Hernández et al. [14] after 1 day of treatment with MnCl_2_ or Citrate of Mn(II) from 0 to 2000 µM.

### 2.4. Transcriptomics

#### 2.4.1. RNA Extraction and Purification

The RA-*SH-SY5Y* cells were treated for 24 h with 500 µM of MnCl_2_ or Mn(II)Cit. Then the cells were collected in RNA*later* (Qiagen, Toronto, ON, Canada), and total RNA was extracted and purified from three different pools of cells using the miRNeasy mini kit (Qiagen, Toronto, ON, Canada), followed by a DNase I (Qiagen, Toronto, ON, Canada) treatment, as directed by the manufacturer. The amount of RNA was quantified using a spectrophotometer, and the quality was assessed using BioAnalyzer equipment (Agilent Technologies, Palo Alto, CA, USA). All RNA samples showed clear 18S and 28S rRNA peaks and demonstrated an RNA integrity number (RIN) level higher than 8.

#### 2.4.2. Microarray Assays

Using an accessible and commercial human gene expression microarray kit, studies on differential gene expression were done in triplicates from three independent biological groups to discover the mode of action of each chemical species of manganese in RA-differentiated *SH-SY5Y* cells. The experiment was carried out in accordance with the manufacturer’s procedures for one-color microarray-based gene expression analysis (Agilent), which are accessible in: http://www.agilent.com/cs/library/usermanuals/Public/G4140-90040_GeneExpression_OneColor_6.9.pdf, accessed on 29 November 2021).

#### 2.4.3. Validation of Toxicogenomics Results through Real-Time Reverse Transcription-PCR (qRT-PCR)

The qRT-PCR assay has been used for identification of gene alterations in previous studies [44,45]. Thus, we used this approach to verify our results about Microarray (item 2.2.2.2). Table 1 shows the primer sequences that were employed. To complete the qRT-PCR experiment, we extracted total RNA from each sample using the Qiagen RNA extraction kit, followed by cDNA production using the iScript cDNA synthesis kit with SYBR green supermix (Bio-Rad, Hercules, CA, USA), as directed by the manufacturer. qRT-PCR was used to quantify mRNA using Rotor-Gene RG-300 from Corbett research [46]. All investigations were carried out in triplicate by three different biological groups.

### 2.5. Bioinformatics and Data Analysis

The results were presented as the mean ± SEM of at least three separate trials. Fitting sigmoidal curves (Hill slope) to concentration–response data of individual replicates and computing the mean of those replicates yielded the LC50. The D’Agostino and Pearson omnibus normality test verified that our data had a normal or statistical distribution. ANOVA (analysis of variance) and Bonferroni’s tests were employed to discover statistically significant differences. GraphPad Prism was used for fitting and statistical analysis (GraphPad 4.0 Software Inc., San Diego, CA, USA). The arrays were examined using Babelomics’ Gene Expression Pattern Analysis Suite (https://babelomics.bioinfo.cipf.es/, accessed on 13 May 2016) [47], which is an integrated web-based pipeline designed for the analysis of data generated in microarray studies. Normalization, grouping, differential gene expression, class prediction, and functional annotation are all included in the suite.

### 2.6. Prediction of Protein–Protein Interaction (PPI) and Gene Ontology (GO) Analysis

A PPI map is a heterogeneous network of proteins connected by interactions as edges. PPI and GO enrichment analyses were performed utilizing data from the current study, the String database (http://string-db.org, accessed on 30 November 2019) [48], and the Comparative Toxicogenomic Database—CTD (http://ctdbase.org/, accessed on 30 November 2019) [49]. Particularly, String discovers cellular pathways that are enriched in a target list of genes, proteins, or metabolites that are not approximated by the original omics data (using hypergeometric testing against either the entire genome or a user-supplied background gene list), allowing for the extraction of strong mechanistic information [48,50].

## 3. Results

Multiple genetic and environmental variables contribute to the onset and progression of Parkinson’s disease, which is related with the degradation of DAergic neurons in the substantia nigra pars compacta [31]. Indeed, epidemiological studies have connected the development of neurodegeneration to Mn toxicity [22,23,24,51,52]. This can be mediated by impairment of protein metabolism, according to findings in nonhuman models [25,34,35]. To test the validity of this hypothesis, we conducted an unbiased toxicogenomics study on Mn-induced acute neurotoxicity in the human model, RA-differentiated *SH-SY5Y*-DAergic cells.

### 3.1. Manganese-Induced Toxicity and Differential Gene Expression in the RA-Differentiated SH-SY5Y-DAergic Cell Model

**Figure 1:**
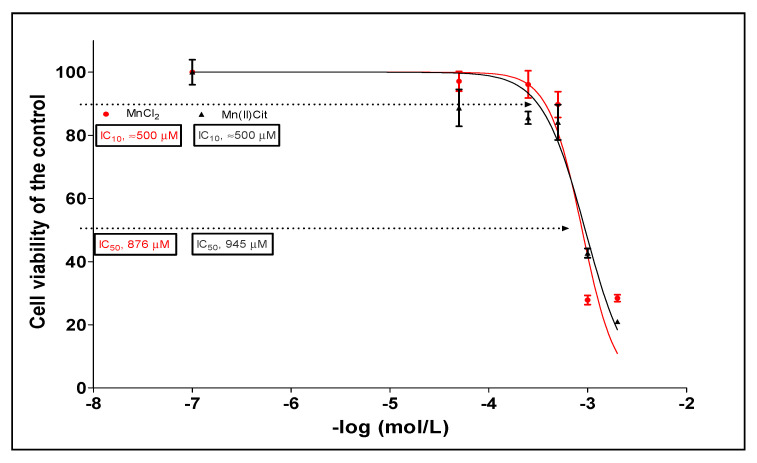
Cytotoxicity induced by MnCl_2_ and Mn(II)Cit in RA-differentiated *SH-SY5Y* cells, after exposure for 24 h. Cell viability was quantified by MTT assay. The plot is representative of three biological replicates (mean ± sem); each experiment was made with five analytical replicates.

Through MTT assay we verified that both Mn species (MnCl_2_ and Mn(II)Cit) induced similar cytotoxicity, in a dose dependent manner, where approximately 900 and 500 µM induces 50% and 10% of cell death, respectively (Figure 1).

**Figure 2:**
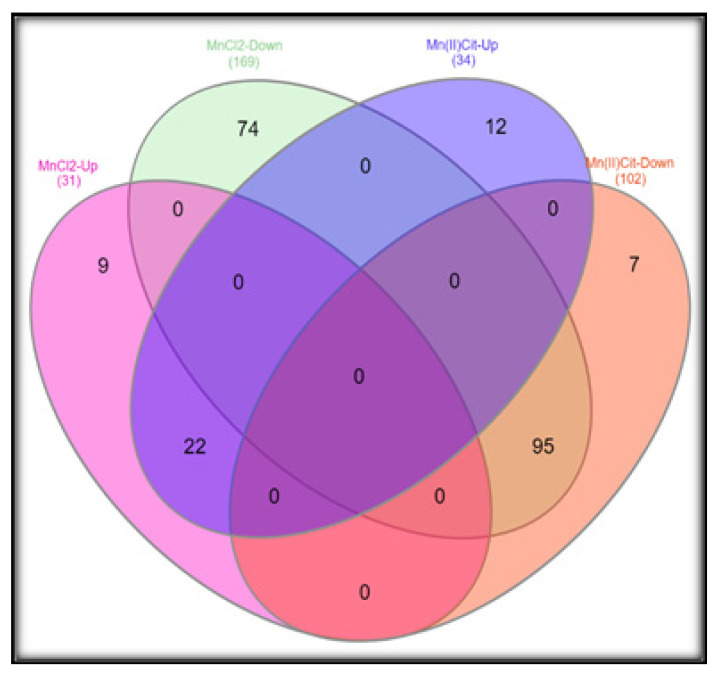
Three microarray assays in *SH-SY5Y* after exposure to MnCl_2_ or Mn(II)Cit (0–500 µM) for 24 h yielded overlapping results of 117 genes differentially expressed and affected by both chemical species of Mn. Significant differences were estimated through two-way ANOVA, followed by false discovery rate and Bonferroni (*p* < 0.05).

To find the mode of action of manganese, we designed a gene expression analysis in *SH-SY5Y* cells treated for 24 h to 500 µM of MnCl_2_ or Mn(II)Cit, which might reflect an environmental state of acute Mn exposure owing to water intake polluted with this metal [15,53,54]. In this way, we verified a significant (*p* < 0.05) impairment of 406 genes (Tables S1 and S2). There, we discovered 117 genes that are affected by both chemical species of manganese, which suppress rather than stimulate gene expression. In this case, MnCl_2_ reduced the expression of 169 genes (80%), while Mn(II)Cit decreased the expression of 102 genes (70%), Figure 2.

### 3.2. Manganese-Induced Cross-Impairment of Several Pathways Associated with Neurotoxicity and Neurodegeneration

**Figure 3:**
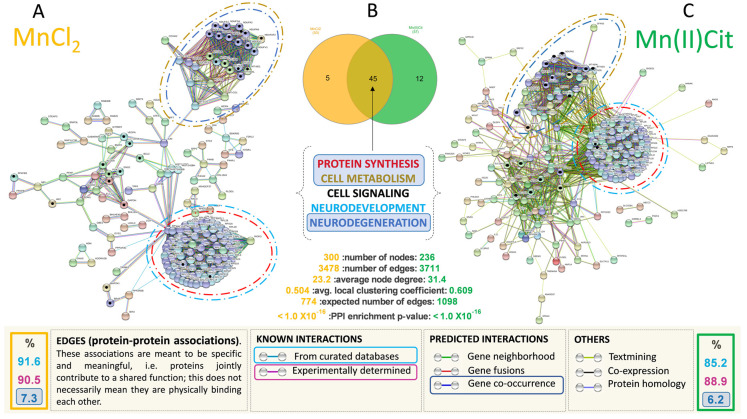
(**A**,**C**) represent an inferred enriched protein–protein interactions (PPI) network for cells stressed with MnCl_2_ and Mn(II)Cit, respectively; *p*-value < 1.0 × 10^−16^. More than 85% of these interactions are derived experimentally. The predicted PPIs were calculated with high confidence score (0.9) and proteins/genes with no connections were removed. (**B**) Overlapping representation of 45 pathways that were affected by MnCl_2_ and Mn(II)Cit. These interactions can be categorized in Protein Synthesis, Cell Metabolism, Cell Signaling, Neurodevelopment, and Neurodegeneration groups.

Additionally, based on all genes significantly affected under Mn stress, and using the String database [48], we used systems biology to create an enriched protein–protein interaction (PPI) map for each Mn species and an enriched gene ontology analysis. The String database studies a defined group of target proteins using physical interactions and functional relationships between proteins, and then expands the set by incorporating linked proteins to investigate toxicological pathways [48]. The network of functional Mn interactors was expanded to around 700 edges (proteins) (*p*-value 1.0 × 10^−16^), with approximately 85 percent of the new interactions experimentally validated [48]. A schematic representation of these interactors is shown in Figure 3A,C, including several processes/pathways potentially impaired by Mn (Table 2 and Table 3).

We identified that almost 50% of the pathways affected by Mn are directly linked to protein biosynthesis, including ribosomes, translation initiation, and termination. Additionally, we verified that MnCl_2_-induced impairment of protein metabolism involves alteration of the metabolism of amino acids. The Mn(II)Cit appears to affect the E3 ubiquitin ligases–target protein degradation pathway, which can lead to damaged/unfolded protein accumulation. This is followed by pathways associated with cell metabolism, especially energy metabolism (~30%) and cell signaling pathways (6%), Table 2 and Table 3. Although the chemical speciation has influenced the Mn-induced toxicity, we found that both species, MnCl_2_ and Mn(II)Cit shared impaired pathways (Figure 3B), which are similar to molecular changes linked with neurodegenerative illnesses such as AD, HD, and PD (Figure 3A,C and Table 2 and Table 3).

**Figure 4:**
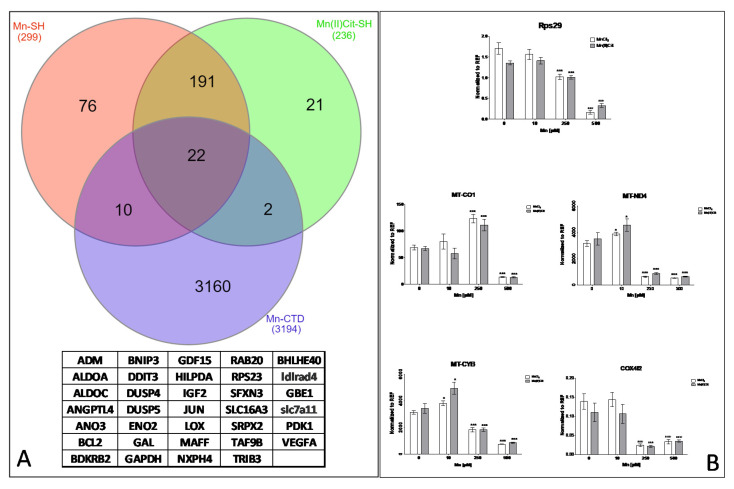
(**A**) The enriched set of proteins identified using PPI data, including genes identified by transcriptomics analysis and added by String were analyzed using the comparative toxicogenomic data. A set of 34 curated genes were identified that previously were affected by MnCl_2_ or other Mn compounds. (**B**) Representative genes associated with protein synthesis, cell metabolism, and neurodegeneration were analyzed by qRT-PCR analysis (mean ± sem, *n* = 3). Statistical differences were verified by two-way ANOVA, followed by Bonferroni post test, (*, *p* < 0.05; ***, *p* < 0.001).

Furthermore, through an additional analysis using CTD [49], among the genes that were directly affected by Mn species (Tables S1 and S2) and the genes/proteins added by String (Figure 3A,C), we identified 34 curated genes (Figure 4A), previously affected by chemical species of Mn, including MnCl_2_. Again, these genes are involved in the pathways inferred in this study (Table 2 and Table 3). Moreover, we confirmed using qRT-PCR analysis that both MnCl_2_ and Mn(II)Cit disrupted the expression of the genes RPS29, MT-CO1, MT-ND4, MT-CYB, and COX4I2, which are associated with protein synthesis, energy metabolism, and neurodegeneration [49] (Figure 4B).

**Figure 5:**
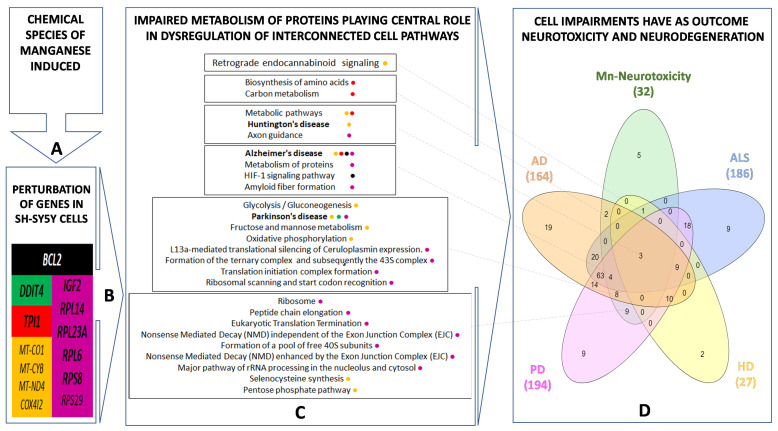
Comparative toxicogenomics analysis for Mn-induced cytotoxicity in *SH-SY5Y*. Exposure of *SH-SYSY* cells to different chemical species of Mn (**A**) results in disruption of genes shown in (**B**). This can lead to impairment of protein metabolism that plays a central role in the dysregulation of several pathways such as cell signaling; cell metabolism, including energy pathway; Alzheimer’s Disease (AD); Huntington’s Disease (HD) and Parkinson’s Disease (PD) (**C**). Overlapping pathways among Mn-induced neurotoxicity and Amyotrophic Lateral Sclerosis (ALS), AD, HD, and PD is shown in (**D**).

Lastly, our findings suggest that chemical species of Mn induce cytotoxicity in the RA-differentiated *SH-SY5Y*-DAergic cell model (Figure 1 and Figure 5A) through alterations of the expression in a notable number of genes (Tables S1 and S2 and Figure 5B), because of which numerous pathways are harmed (Figure 3 and Figure 5C). There, disruption of protein metabolism, especially protein synthesis, appears to be a key event for Mn-induced cell perturbation (Figure 5C) responsible for Mn-induced neurotoxicity, which may result in neurodegeneration (Figure 5D).

## 4. Discussion

In this work, we found that the 876 µM of MnCl_2_ or 945 µM of Mn(II)Cit decreased by 50% the viability of RA-differentiated *SH-SY5Y*-DAergic cells. These concentrations are higher than found in studies with NoRA-*SH-SY5Y* cells, where 800 µM [55] and approximately 600 µM [40] of MnCl_2_, respectively, induced 50% of cytotoxicity after exposure for 24 h. It is known that RA-differentiated *SH-SY5Y* cells show activated survival pathways, including Nrf2 (nuclear factor erythroid 2-related factor 2) or Akt (serine-threonine protein kinase) signaling [29], while the NoRA-differentiated cells are ATP-deficient compared to the RA-differentiated cells [31]. This could justify why NoRa *SH-SY5Y* cells are more susceptible for Mn treatment, as observed by Kemsheh and Oblitey [56]. On the other hand, the RA-differentiated *SH-SY5Y*-DAergic cell model shows increased dopamine and other neurotransmitters, which allowed the screening of Mn-induced neurotoxicity and their association with neurodegeneration [31,32,33,57], after exposure for 500 µM of both Mn-species, a concentration that induced approximately 10% of cell death and was used in previous studies with NoRA-differentiated cells [58]. In this toxicologic context, the CTD have systematized approximately 140 Mn compounds associated with 379 pathways [49], where the top fifty are dominated by eleven cellular processes, including cell signaling, diseases, immune system, metabolism, hemostasis, metabolism of proteins, apoptosis, endocrine resistance, cellular responses to stress, and neurodevelopment. These processes are potentially connected to 1522 diseases, of which 21 are associated with the nervous system [49]. Other functional and omics studies [25,26,27,28,34,35] have identified these potential mechanisms of Mn-induced neurotoxicity, reviewed by Tinkov et al. [52]. In line with this, using microarray analysis and systems biology approaches, we verified a connection between Mn-induced acute cytotoxicity in the RA-differentiated *SH-SY5Y*-DAergic cell model and impairment of several pathways, including protein synthesis (18 processes), cell metabolism (10 processes), cell signaling (3 processes), neurodevelopment (1 process), and neurodegeneration (3 processes) (Table 2 and Table 3).

Presynaptic local protein synthesis, the process by which mRNAs are translated in axons and terminals, is well shown to be mediated by retrograde endocannabinoid (eCB) transmission [59]. The eCBs are lipids that are mobilized by postsynaptic action and travel backward across the synapse to bind presynaptic Gi/o-coupled type 1 cannabinoid (CB1) receptors, inhibiting neurotransmitter release. CB1 activation, in turn, boosts protein synthesis via the mTOR pathway [59]. Indeed, we identified impairment of retrograde endocannabinoid signaling, which can contribute to the disruption of axon guidance, after exposure to Mn (Table 2 and Table 3). Furthermore, it has been proposed that chemical stresses can either upregulate or downregulate the endocannabinoid system. This can disrupt synaptic transmission and brain circuit functions [60], leading to neurodegenerative illnesses such as AD, ALS, HD, and PD [61,62], as well as activate other signaling systems [60] such as hypoxia-inducible factor 1 (HIF-1), whose activation contributes to recovery of synaptic functions [63]. However, depending on the chemical speciation and the biological model investigated, HIF-1 can be inactivated due to downregulation of the BCL2 gene in response to Mn stress. Mn deficiency reduces the production of BCL2 mRNA and protein [64]. Manganese (III)-tetrakis(4-benzoic acid)porphyrin has been shown in studies with isolated human endothelial cells to inhibit the decrease of protein BCL2 expression [65]. Exposure to maneb and mancozeb, both Mn-based pesticides, results in increased expression of *BCL2* mRNA and BCL2 protein abundance [66,67].

In agreement with our findings (Figure 3 and Figure 5, Table 2 and Table 3), researchers discovered altered genes related to neurogenesis, neurodevelopment, synaptic transmission, and apoptosis after exposing NoRa-differentiated SH-SY5Y cells to 100 µM MnCl_2_ for 30 days, which may be linked to neurodegeneration [26]. However, this work did not identify that these alterations can be preceded by impaired protein metabolism. Indeed, before changes in the mitochondrial–energy pathway and neurotransmission system, Fernandes et al. discovered increased abundance of the genes BET1 (Golgi vesicular membrane–trafficking protein), ADAM10 (ADAM metallopeptidase domain 10), and ARFGAP3 (ADP-ribosylation factor GTPase-activating protein 3) [28]. Accordingly, we confirmed that 500 µM of Mn(II)Cit or MnCl_2_ caused neurotoxicity in RA-differentiated SH-SY5Y-DAergic cells by disrupting approximately 50% of protein metabolism-related pathways, followed by energy dysfunction and other related molecular cell changes (Table 2 and Table 3). This is consistent with previous observations that MnCl_2_-induced neurotoxicity in NoRA SH-SY5Y was caused by changes in the ER–Golgi complex [38,39]. Hernández’s group’s research with a variety of models ranging from yeast to mammals, different Mn-species, and different exposure times has also suggested that impaired protein metabolism is a key event in Mn-induced neurotoxicity [25,34,35].

Although RA-differentiated *SH-SY5Y* can have altered axons [29,30], certainly Mn induces disturbance of axonal functions potentially linked to protein synthesis impairment [68]. Studies with primary cerebellar granule neurons exposed for MnCl_2_ identified potential axonal alteration due to hypokalemia and overexpression of STX1A, implicated in the production of presynaptic local proteins that regulate neurotransmitter release [25]. Here, in RA-differentiated SH-SY5Y exposed for Mn, we identified significant alterations of inferred and curated genes such as *IGF2*, *RPL14*, *RPL23A*, *RPL6*, *RPS8,* and *RPS29* (Figure 2B and Figure 4D), transcripts hit revealed using the String database [48] and CTD [49]. These genes are involved in an enriched set of pathways linked to translation, post-translation modifications, and protein degradation (Figure 3A,C, Table 2 and Table 3), that can be perturbed by disruption of energy pathways [36,37] as well. Here, we found that the species of Mn can increase or arrest the expression of genes such as *MT-CO1*, *MT-CYB*, *MT-ND4,* and *COX4I2* (Figure 4B), which are associated with glycolysis and glycogenesis, and oxidative phosphorylation, along with neurodegenerative diseases such as AD, HD, and PD [49]. Concomitantly, we identified impairment of *TPI1* gene, which is associated with one-carbon metabolism pathway, biosynthesis of amino acid, and AD [49]. Furthermore, disruption of amino acid biosynthesis has been linked to the alteration of translation efficiency [69], similar to that observed in yeast exposed to MnCl_2_ for 24 h, which showed decreased β-galactosidase reporter expression, heavy polysomes fractions, and the expression of *NOP1* and *NSR1* genes, associated with ribosome biogenesis [34]. Altogether, the *RPL14* gene impaired by Mn(II) is a marker of PD, whose alteration has been detected already, after domestic exposure to maneb (manganese(2+);*N*-[2-(sulfidocarbothioylamino)ethyl]carbamodithioate) [70]. We have discovered maneb-impaired protein metabolism in cerebellar granule neurons [25]. Another study identified Mn-induced ER-stress and increased phosphorylation of translation initiation factor *eIF2α* in *SH-SY5Y* cells [70]. Collectively, these findings confirm that the network among protein metabolism, energy, cell development, and metabolic pathways [36,37] may be disrupted by chemical species of Mn [34,35,71,72,73,74,75].

Contrary to the transcriptomics studies conducted by Gandhi et al. [26] and Fernandes et al. [28], which considered only MnCl_2_, we verify that chemical speciation is important for Mn-induced impairment of gene expression and protein metabolism on RA-differentiated *SH-SY5Y* cells. For example, the MnCl_2_-induced alteration of the metabolism of amino (AA) acids, which influences RNA metabolism and protein synthesis [76], similar to that identified in yeast exposed for MnCl_2_ [34]. It has been demonstrated that amino acids are a regulator of late endosomes/lysosomes anterograde transport, which operate as mRNA translation platforms to produce new proteins necessary to support mitochondria function in axons growth, which is sensible to energy stress [77]. Indeed, we verified cross-talk alterations of the metabolism of AAs, proteins, cell energy, and axon guidance in response to cytotoxic concentrations of Mn. A metabolomics study with NoRA *SH-SY5Y* cells exposed to noncytotoxic concentrations of MnCl_2_ revealed a positive association between Mn exposure and glutamate and *N*-acetylglutamate semialdehyde, and a negative relationship with other AAs such as leucine/isoleucine, 4-imidazoleacetate, histidine, arginine, and valine. As a result, neurotransmitter-related metabolites such as GABA, adrenochrome, N4-acetylaminobutanal, N-methyl salsolinol, and dopamine sulfate were dramatically altered [27]. This corroborates the importance of the AAs for local protein synthesis, during neurodevelopment, which can be impaired by Mn.

Although RA can be anti-proteasome inhibitor in SH-SY5Y cells [33], we deduced that Mn(II)Cit induces neurotoxicity associated with impairment of E3 ubiquitin ligases–target protein degradation pathway, which is involved in the proteasome’s identification of substrates and proteins for degradation [78]. This can lead to damaged/unfolded protein accumulation, consistent with altered expression of the genes *HIST1H2BB, HIST1H2BH,* and *HIST1H2BO* (Figure 3A,C). Histone modification is linked to the creation of amyloid fibers (Table 3) and, as a result, the development of neurodegenerative diseases [79]. Other research has shown that Mn-induced neurotoxicity can target the ubiquitin system. For example, in cultured astrocytes treated with MnCl_2_, SNAT3 protein degradation and Gln homeostasis disruption occur via the ubiquitin-mediated proteolytic mechanism [80]. Additionally, in cerebellar granule cells stressed with MnCl_2_ or maneb, the Mn induces impairment of protein metabolism, involving dysregulation of the ubiquitin system as well [25]. However, the ubiquitin system was not altered in dopaminergic cells (SH-SY5Y and CATH.a), with impaired ER–Golgi complex, under the effect of Mn [39].

## 5. Conclusions

Lastly, we identified some advantages to using the RA-differentiated cells instead of the undifferentiated *SH-SY5Y* cell line to study Mn-induced neurotoxicity in humans. These cells are chemically and genetically modified; consequently, it cannot be considered normal [31], which might restrict the experiment’s outcomes in a variety of ways. Our findings, however, are equivalent to those obtained with primary culture of mouse cerebellar granule neurons. At the same time, we revealed that RA-differentiated *SH-SY5Y* cells respond differently to distinct chemical species of Mn, which was not considered in previous studies with NoRA-differentiated *SH-SY5Y* cells. Indeed, Mn causes disruption of cross-talk networks of pathways in the RA-differentiated *SH-SY5Y*-DAergic cells, which may be mediated by protein metabolism disturbance, evidently influenced by chemical speciation, for example, the MnCl_2_-altered amino acid metabolism, which affects RNA metabolism and protein synthesis. Mn(II)Cit altered the E3 ubiquitin ligases–target protein degradation pathway, potentially leading to the accumulation of damaged/unfolded proteins, which is consistent with histone modification. These findings support the relevance of chemical speciation in understanding the process behind Mn-induced neurotoxicity and neurodegeneration, which appears to be conserved from yeast [34], to zebrafish [35], to mammals [25,28]. A functional analysis of cross-species translation in the presence of Mn could either confirm or refute our findings.

## Figures and Tables

**Table 1:** Genes selected for qRT-PCR analysis. Primer sequences and associated parameters are included.

Gene Symbol	Gene Name	PL	Sequence (5′→3′)		TS	Length	Start	Stop	Tm	GC%	Self C.	Self 3′ C.
RPS29	RPS29 ribosomal protein S29	135	FP	ACACTGGCGGCACATATTGA	Plus	20	49,585,986	49,586,005	60.04	50	4	2
			RP	GGTAGTAGCCGTCTGAGTGC	Minus	20	49,586,120	49,586,101	59.9	60	4	3
MT-CO1	mitochondrially encoded cytochrome c oxidase I	107	FP	CCCCGATGCATACACCACAT	Plus	20	7232	7251	60.18	55	6	2
			RP	TCGAAGCGAAGGCTTCTCAA	Minus	20	7338	7319	59.68	50	7	3
COX4I2	cytochrome c oxidase subunit 4I2	118	FP	GATGAACCGTCGCTCCAATG	Plus	20	30,135,127	30,135,146	59.35	55	5	3
			RP	GATGAGGTGTTGCCACTCAC	Minus	20	30,135,244	30,135,225	58.84	55	4	3
MT-CYB	mitochondrially encoded cytochrome b	134	FP	ACCCCCTAGGAATCACCTCC	Plus	20	15,366	15,385	60.03	60	6	1
			RP	GCCTAGGAGGTCTGGTGAGA	Minus	20	15,499	15,480	60.03	60	6	2
MT-ND4	mitochondrially encoded NADH dehydrogenase 4	296	FP	CCCCATCGCTGGGTCAATAG	Plus	20	11,428	11,447	60.25	60	8	2
			RP	TAAGCCCGTGGGCGATTATG	Minus	20	11,723	11,704	60.25	55	6	1
Gapdh	glyceraldehyde-3-phosphate dehydrogenase	117	FP	AAAGGGCCCTGACAACTCTTT	Plus	21	6,538,069	6,538,089	59.78	47.62	8	3
			RP	GGTGGTCCAGGGGTCTTACT	Minus	20	6,538,185	6,538,166	60.55	60	5	1

Product Length (PL), Forward primer (FP), Reverse primer (RP), Template Strand (TS), Self Complementarity (Self C.), Self 3′ Complementarity (Self 3′ C.).

**Table 2:** Enriched cellular pathways influenced by MnCl_2_ in RA-differentiated *SH-SY5Y* cells, after gene ontology analysis using the String database.

Cell Pathways	Term Description	Observed	Background	FDR
		Gene Count	Gene Count	
Protein Synthesis	Peptide chain elongation	75	86	4.37 × 10^−92^
	Viral mRNA Translation	75	86	4.37 × 10^−92^
	SRP-dependent cotranslational protein targeting to membrane	78	109	1.15 × 10^−91^
	Eukaryotic Translation Termination	75	90	1.22 × 10^−91^
	Nonsense Mediated Decay (NMD) independent of the Exon Junction Complex (EJC)	75	92	2.25 × 10^−91^
	Formation of a pool of free 40S subunits	75	98	5.96 × 10^−90^
	L13a-mediated translational silencing of Ceruloplasmin expression	75	107	5.93 × 10^−88^
	GTP hydrolysis and joining of the 60S ribosomal subunit	75	108	9.04 × 10^−88^
	Ribosome	76	130	3.15 × 10^−84^
	Nonsense Mediated Decay (NMD) enhanced by the Exon Junction Complex (EJC)	75	112	6.29 × 10^−87^
	Major pathway of rRNA processing in the nucleolus and cytosol	76	179	1.10 × 10^−76^
	Metabolism of amino acids and derivatives	77	354	2.98 × 10^−59^
	Metabolism of RNA	78	652	7.72 × 10^−43^
	Formation of the ternary complex and subsequently the 43S complex	31	49	1.27 × 10^−34^
	Translation initiation complex formation	31	55	1.85 × 10^−33^
	Ribosomal scanning and start codon recognition	31	55	1.85 × 10^−33^
	Metabolism of proteins	102	1948	1.12 × 10^−27^
	Biosynthesis of amino acids	9	72	5.98 × 10^−05^
Cell Metabolism	Selenocysteine synthesis	75	90	1.22 × 10^−91^
	Selenoamino acid metabolism	76	112	2.37 × 10^−88^
	Metabolism	137	2032	6.11 × 10^−52^
	Oxidative phosphorylation	26	131	9.48 × 10^−18^
	The citric acid (TCA) cycle and respiratory electron transport	28	173	7.83 × 10^−18^
	Thermogenesis	29	228	1.68 × 10^−15^
	Complex I biogenesis	17	55	8.42 × 10^−15^
	Metabolic pathways	50	1250	2.11 × 10^−08^
	Glycolysis/Gluconeogenesis	12	68	5.27 × 10^−08^
	Fructose and mannose metabolism	9	33	1.85 × 10^−07^
	Metabolism of carbohydrates	20	266	2.71 × 10^−07^
	Carbon metabolism	10	116	2.90 × 10^−04^
Cell Signaling	Retrograde endocannabinoid signaling	18	148	2.25 × 10^−09^
	HIF-1 signaling pathway	14	98	3.22 × 10^−08^
	Negative regulation of MAPK pathway	6	40	1.00 × 10^−03^
Neurodevelopment	Axon guidance	79	541	3.00 × 10^−49^
Neurodegeneration	Parkinson’s disease	26	142	3.69 × 10^−17^
	Alzheimer’s disease	20	168	3.22 × 10^−10^
	Huntington’s disease	19	193	1.57 × 10^−08^

False Discovery Rate—FDR.

**Table 3:** Enriched cellular pathways influenced by Mn(II)Cit in RA-differentiated *SH-SY5Y* cells, after gene ontology analysis using String-db.

Cell Pathways	Term Description	Observed	Background	FDR
		Gene Count	Gene Count	
Protein Synthesis	Peptide chain elongation	76	86	7.69 × 10^−103^
	Viral mRNA Translation	76	86	7.69 × 10^−103^
	Eukaryotic Translation Termination	76	90	2.96 × 10^−102^
	Nonsense Mediated Decay (NMD) independent of the Exon Junction Complex (EJC)	76	92	4.89 × 10^−102^
	Formation of a pool of free 40S subunits	76	98	1.34 × 10^−100^
	L13a-mediated translational silencing of Ceruloplasmin expression	76	107	1.58 × 10^−98^
	GTP hydrolysis and joining of the 60S ribosomal subunit	76	108	2.37 × 10^−98^
	SRP-dependent cotranslational protein targeting to membrane	76	109	3.59 × 10^−98^
	Nonsense Mediated Decay (NMD) enhanced by the Exon Junction Complex (EJC)	76	112	1.52 × 10^−97^
	Ribosome	77	130	6.14 × 10^−95^
	Formation of the ternary complex and subsequently the 43S complex	31	49	5.38 × 10^−38^
	Translation initiation complex formation	31	55	7.97 × 10^−37^
	Ribosomal scanning and start codon recognition	31	55	7.97 × 10^−37^
	Metabolism of proteins	88	1948	2.38 × 10^−27^
	Biosynthesis of amino acids	8	72	6.26 × 10^−05^
	Senescence-Associated Secretory Phenotype (SASP)	6	78	6.50 × 10^−03^
	Amyloid fiber formation	6	78	6.50 × 10^−03^
	E3 ubiquitin ligases ubiquitinate target proteins	5	53	7.80 × 10^−03^
Cell Metabolism	Selenocysteine synthesis	76	90	2.96 × 10^−102^
	Oxidative phosphorylation	26	131	2.08 × 10^−20^
	Thermogenesis	28	228	2.41 × 10^−17^
	Metabolic pathways	45	1250	1.71 × 10^−09^
	Glycolysis/Gluconeogenesis	12	68	3.66 × 10^−09^
	Carbon metabolism	10	116	3.67 × 10^−05^
	Pentose phosphate pathway	5	30	6.60 × 10^−04^
	Starch and sucrose metabolism	5	33	9.30 × 10^−04^
	Galactose metabolism	4	31	8.10 × 10^−03^
	Endocrine resistance	6	95	1.26 × 10^−02^
Cell Signaling	Retrograde endocannabinoid signaling	16	148	2.85 × 10^−09^
	HIF-1 signaling pathway	12	98	1.42 × 10^−07^
	Negative regulation of MAPK pathway	5	40	2.50 × 10^−03^
Neurodevelopment	Axon guidance	78	541	5.39 × 10^−57^
Neurodegeneration	Parkinson’s disease	26	142	8.36 × 10^−20^
	Alzheimer’s disease	20	168	3.71 × 10^−12^
	Huntington’s disease	19	193	2.88 × 10^−10^

False Discovery Rate—FDR.

## Data Availability

Not applicable.

## Supplementary Materials

The following are available online at https://www.mdpi.com/article/10.3390/toxics9120348/s1, Table S1: Genes differentially expressed in RA-differentiated *SH-SY5Y* cells after exposure to MnCl_2_ are listed below. Table S2: Genes differentially expressed in RA-differentiated *SH-SY5Y* cells after exposure to MnCl_2_ are listed below.
