# Supplementary material for: Manganese-Induced Neurotoxicity through Impairment of Cross-Talk Pathways in Human Neuroblastoma Cell Line SH-SY5Y Differentiated with Retinoic Acid
Source: Toxics. 2021 Dec 9;9(12):348. doi: 10.3390/toxics9120348 (PMC8704659; doi:10.3390/toxics9120348)
Supplement: Supplementary file 1 [file toxics-09-00348-s001.zip › toxics-1429145-supplementary.pdf]

toxics MDPI

Supplementary Materials: Manganese-Induced Neurotoxicity through Impairment of Cross-Talk Pathways in Human Neuroblastoma Cell Line *SH-SY5Y* Differentiated with Retinoic Acid

Raúl Bonne Hernández, Nadja C. de Souza-Pinto, Jos Kleinjans, Marcel van Herwijnen, Jolanda Piepers, Houman Moteshareie, Daniel Burnside and Ashkan Golshani

Table S1. Genes differentially expressed in RA-differentiated SH-SY5Y cells after exposure to $MnCl_2$ are listed below.

Chemical Specie	Effect	Gene Symbol	Gene Name
$MnCl_2$	Up-expression	RASL11B	Ras-like protein family member 11B
		SCLY	Selenocysteine lyase
		ABCA12	ATP-binding cassette sub-family A member 12
		CORIN	Atrial natriuretic peptide-converting enzyme
		GPD1L	Glycerol-3-phosphate dehydrogenase 1-like protein
		NDUFAF2	NADH dehydrogenase [ubiquinone] 1 alpha subcomplex assembly factor 2
		RAC3	Ras-related C3 botulinum toxin substrate 3
		TAF9B	Transcription initiation factor TFIID subunit 9B
		WLS	Protein wntless homolog
		MT-CO1	Cytochrome c oxidase subunit 1
		MT-CYB	Cytochrome b
		MT-ND1	NADH-ubiquinone oxidoreductase chain 1
		MT-ND5	NADH-ubiquinone oxidoreductase chain 5
		MT-ND4	NADH-ubiquinone oxidoreductase chain 4
		NTNG1	Netrin-G1
		ROR1	Inactive tyrosine-protein kinase transmembrane receptor ROR1
		TRIM63	E3 ubiquitin-protein ligase TRIM63
		C6orf48	Protein G8
		ZNF157	Zinc finger protein 157
		AHNAK	Neuroblast differentiation-associated protein AHNAK
		STEAP3	Metalloreductase STEAP3
		RPS29	Ribosomal protein S29
		BCL2	Apoptosis regulator Bcl-2
		WDR4	tRNA (guanine-N(7)-)-methyltransferase non-catalytic subunit WDR4
		CCNL2	Cyclin-L2
		SCFD2	Sec1 family domain-containing protein 2
		KIF26B	Kinesin-like protein KIF26B
		CHST15	Carbohydrate sulfotransferase 15
		KIRREL3	Kin of IRRE-like protein 3
		RAB40B	Ras-related protein Rab-40B
		COLCA2	Colorectal cancer associated 2
	Down-expression	WDR54	WD repeat-containing protein 54
		PPP2R5B	Serine/threonine-protein phosphatase 2A 56 kDa regulatory subunit beta isoform
		C4orf6	Uncharacterized protein encoded by LINC01587
		PLOD1	Procollagen-lysine
		CABP7	Calcium-binding protein 7
		TRIB3	Tribbles homolog 3
		SFXN3	Sideroflexin-3

		DUSP3	Dual specificity protein phosphatase 3
		ALDOC	Aldolase
		GALNT18	Polypeptide N-acetylgalactosaminyltransferase 18
		GAPDH	Glyceraldehyde-3-phosphate dehydrogenase
		TPI1	Triosephosphate isomerase 1
		HBEGF	Proheparin-binding EGF-like growth factor
		LOX	Protein-lysine 6-oxidase
		PFKFB4	6-phosphofructo-2-kinase/fructose-2
		ENO1	Alpha-enolase
		ELL2	RNA polymerase II elongation factor ELL2
		PPP1R3C	Protein phosphatase 1 regulatory subunit 3C
		DUSP4	Dual specificity protein phosphatase 4
		KIAA1045	PHD finger protein 24
		BHLHE41	Class E basic helix-loop-helix protein 41
		SH3TC1	SH3 domain and tetratricopeptide repeats 1
		INSIG2	Insulin-induced gene 2 protein
		GDF15	Growth differentiation factor 15
		NTS	Neurotensin/neuromedin N
		BHLHE40	Class E basic helix-loop-helix protein 40
		ANO3	Anoctamin-3
		EMP1	Epithelial membrane protein 1
		HNF1A	Hepatocyte nuclear factor 1-alpha
		HILPDA	Hypoxia-inducible lipid droplet-associated protein
		CCL27	C-C motif chemokine 27
		IER3	Radiation-inducible immediate-early gene IEX-1
		HS3ST2	Heparan sulfate glucosamine 3-O-sulfotransferase 2
		P4HA1	Prolyl 4-hydroxylase subunit alpha-1
		NGEF	Ephexin-1
		LOXL3	Lysyl oxidase homolog 3
		IL11	Interleukin-11
		HRASLS	Phospholipid-metabolizing enzyme A-C1
		STC2	Stanniocalcin-2
		ABCB6	ATP-binding cassette sub-family B member 6
		GAL	Galanin peptides
		MTFP1	Mitochondrial fission process protein 1
		GABARAPL1	Gamma-aminobutyric acid receptor-associated protein-like 1
		RAB20	Ras-related protein Rab-20
		MAP1LC3B	Microtubule-associated proteins 1A/1B light chain 3B
		PPFIA4	Liprin-alpha-4
		KIAA1715	Endoplasmic reticulum junction formation protein lunapark
		ZNF215	Zinc finger protein 215
		RAB6B	Ras-related protein Rab-6B
		MAGEC1	Melanoma-associated antigen C1
		C8orf58	Uncharacterized protein C8orf58
		HK2	Hexokinase-2
		PDXK	Pyridoxal kinase
		PRSS12	Neurotrypsin
		SCRG1	Scrapie-responsive protein 1
		F2RL2	Proteinase-activated receptor 3
		MICALL2	MICAL-like protein 2
		ANGPTL4	Angiopoietin-related protein 4
		MAP2K1	Dual specificity mitogen-activated protein kinase kinase 1
		GTPBP2	GTP binding protein 2
		ADORA2B	Adenosine receptor A2b

		ISG20	Interferon-stimulated gene 20 kDa protein
		DDIT4	DNA damage-inducible transcript 4 protein
		RIN1	Ras and Rab interactor 1
		FAM57A	Protein FAM57A
		CHRNA9	Neuronal acetylcholine receptor subunit alpha-9
		TNIP1	TNFAIP3-interacting protein 1
		GYS1	Glycogen [starch] synthase
		MPI	Mannose-6-phosphate isomerase
		AQP10	Aquaporin-10
		TMEM45A	Transmembrane protein 45A
		PHLDA2	Pleckstrin homology-like domain family A member 2
		ANKZF1	Ankyrin repeat and zinc finger domain-containing protein 1
		PGA3	Pepsin A-3
		EBF1	Transcription factor COE1
		BAIAP3	BAI1 associated protein 3
		P4HB	Protein disulfide-isomerase
		ARHGEF37	Rho guanine nucleotide exchange factor 37
		CYB5D1	Cytochrome b5 domain containing 1
		ASCL2	Achaete-scute homolog 2
		ZNF470	Zinc finger protein 470
		NXPH4	Neurexophilin-4
		RAX	Retinal homeobox protein Rx
		EDARADD	Ectodysplasin-A receptor-associated adapter protein
		ANKRD37	Ankyrin repeat domain-containing protein 37
		ANG	Angiogenin
		RIOK3	Serine/threonine-protein kinase RIO3
		STMN4	Stathmin-4
		ATF3	Cyclic AMP-dependent transcription factor ATF-3
		DHRS2	Dehydrogenase/reductase SDR family member 2
		MAFF	Transcription factor MafF
		MYH7	Myosin-7
		DOK3	Docking protein 3
		SERTAD1	SERTA domain-containing protein 1
		GOLGA8A	Golgin subfamily A member 8A
		AKR1B10	Aldo-keto reductase family 1 member B10
		MAGEC2	Melanoma-associated antigen C2
		MT-ND6	NADH-ubiquinone oxidoreductase chain 6
		RHOU	Rho-related GTP-binding protein RhoU
		LEMD1	LEM domain containing 1
		FAM129A	Protein Niban
		BNIP3	BCL2/adenovirus E1B 19 kDa protein-interacting protein 3
		DUSP5	Dual specificity protein phosphatase 5
		DPCD	Protein DPCD
		PGAM1	Phosphoglycerate mutase 1
		PGM1	Phosphoglucomutase-1
		GNAS	Guanine nucleotide-binding protein G(s) subunit alpha isoforms XLas
		BEND5	BEN domain-containing protein 5
		IL13RA2	Interleukin-13 receptor subunit alpha-2
		FUT11	Alpha-(1
		SRPX2	Sushi repeat-containing protein SRPX2
		PGK1	Phosphoglycerate kinase 1
		SPAG4	Sperm-associated antigen 4 protein
		COX4I2	Cytochrome c oxidase subunit 4 isoform 2
		PTPRH	Receptor-type tyrosine-protein phosphatase H

		HIST1H2BL	Histone H2B type 1-L
		CA9	Carbonic anhydrase 9
		ID4	DNA-binding protein inhibitor ID-4
		C4orf47	UPF0602 protein C4orf47
		TUBB2A	Tubulin beta-2A chain
		BNIP3L	BCL2/adenovirus E1B 19 kDa protein-interacting protein 3-like
		PFKP	ATP-dependent 6-phosphofructokinase
		PDK1	[Pyruvate dehydrogenase (acetyl-transferring)] kinase isozyme 1
		GAP43	Neuromodulin
		NDUFA4L2	NADH dehydrogenase [ubiquinone] 1 alpha subcomplex subunit 4-like 2
		VKORC1	Vitamin K epoxide reductase complex subunit 1
		SPP1	Osteopontin
		ALDOA	Fructose-bisphosphate aldolase A
		AK4	Adenylate kinase 4
		ODAM	Odontogenic ameloblast-associated protein
		GPR146	Probable G-protein coupled receptor 146
		TSPAN4	Tetraspanin-4
		CEP250	Centrosome-associated protein CEP250
		IRF7	Interferon regulatory factor 7
		COL13A1	Collagen alpha-1(XIII) chain
		ENSG00000164096	Uncharacterized protein C4orf3
		P4HA2	Prolyl 4-hydroxylase subunit alpha-2
		PDK3	[Pyruvate dehydrogenase (acetyl-transferring)] kinase isozyme 3
		SEC61G	Protein transport protein Sec61 subunit gamma
		SEMA5B	Semaphorin-5B
		IGF2	Insulin-like growth factor II
		TTC39A	Tetratricopeptide repeat domain containing
		PDZK1	Na(+)/H(+) exchange regulatory cofactor NHE-RF3
		PPP1R13L	RelA-associated inhibitor
		NDRG1	Protein NDRG1
		GBE1	1, 4-alpha-glucan-branching enzyme 1
		RIMKLA	N-acetylaspartylglutamate synthase A
		SLC2A1	Solute carrier family 2
		FAM162A	Protein FAM162A
		TFR2	Transferrin receptor protein 2
		SH3D21	SH3 domain containing 21
		MPP2	MAGUK p55 subfamily member 2
		LGALS8	Galectin 8
		ADM	ADM
		ENO2	Gamma-enolase
		PFKFB3	6-phosphofructo-2-kinase/fructose-2
		LDHA	Lactate dehydrogenase A
		DDIT3	DNA damage-inducible transcript 3 protein
		BDKRB2	B2 bradykinin receptor
		GPR56	Adhesion G-protein coupled receptor G1
		LOC730098	HCG2040265
		CCDC64B	BICD family-like cargo adapter 2
		SLC16A3	Monocarboxylate transporter 4
		HIST1H2BO	Histone H2B type 1-O
		MTHFD1L	Monofunctional C1-tetrahydrofolate synthase
		VEGFA	Vascular endothelial growth factor A
		HIST1H2BH	Histone H2B type 1-H
		NUDT18	8-oxo-dGDP phosphatase NUDT18
		HIST1H2BB	Histone H2B type 1-B

Table S2. Genes differentially expressed in RA-differentiated SH-SY5Y cells after exposure to $MnCl_2$ are listed below.

Chemical Specie	Effect	Gene Symbol	Gene Name
Mn(II)Cit	Up-expression	RASL11B	Ras-like protein family member 11B
		RAC2	Ras-related C3 botulinum toxin substrate 2
		ABCA12	ATP-binding cassette sub-family A member 12
		CORIN	Atrial natriuretic peptide-converting enzyme
		GPD1L	Glycerol-3-phosphate dehydrogenase 1-like protein
		KCNE2	Potassium voltage-gated channel subfamily E member 2
		RAC3	Ras-related C3 botulinum toxin substrate 3
		TBC1D16	TBC1 domain family member 16
		MPPED2	Metallophosphoesterase MPPED2
		LDLRAD4	Low-density lipoprotein receptor class A domain-containing protein 4
		NTRK3	NT-3 growth factor receptor
		MT-CO1	Cytochrome c oxidase subunit 1
		MT-CYB	Cytochrome b
		MT-ND5	NADH-ubiquinone oxidoreductase chain 5
		MT-ND4	NADH-ubiquinone oxidoreductase chain 4
		BRINP3	BMP/retinoic acid-inducible neural-specific protein 3
		NTNG1	Netrin-G1
		ROR1	Inactive tyrosine-protein kinase transmembrane receptor ROR1
		FNDC5	Fibronectin type III domain-containing protein 5
		HSD17B8	Estradiol 17-beta-dehydrogenase 8
		C6orf48	Protein G8
		ZNF157	Zinc finger protein 157
		AHNAK	Neuroblast differentiation-associated protein AHNAK
		SOHLH2	Spermatogenesis- and oogenesis-specific basic helix-loop-helix-containing protein 2
		STEAP3	Metalloreductase STEAP3
		RPS29	Ribosomal protein S29
		BCL2	Apoptosis regulator Bcl-2
		KIF26B	Kinesin-like protein KIF26B
		LETMD1	LETM1 domain-containing protein 1
		CHST15	Carbohydrate sulfotransferase 15
		KIRREL3	Kin of IRRE-like protein 3
		RAB40B	Ras-related protein Rab-40B
		TMC6	Transmembrane channel-like protein 6
		COLCA2	Colorectal cancer associated 2
	Down-expression	PLOD1	Procollagen-lysine
		C22orf31	Uncharacterized protein C22orf31
		CABP7	Calcium-binding protein 7
		SFXN3	Sideroflexin-3
		ALDOC	Aldolase
		TPI1	Triosephosphate isomerase 1
		HBEGF	Proheparin-binding EGF-like growth factor
		LOX	Protein-lysine 6-oxidase
		PFKFB4	6-phosphofructo-2-kinase/fructose-2
		ELL2	RNA polymerase II elongation factor ELL2
		PPP1R3C	Protein phosphatase 1 regulatory subunit 3C
		DUSP4	Dual specificity protein phosphatase 4
		INSIG2	Insulin-induced gene 2 protein
		GDF15	Growth differentiation factor 15
		ANO3	Anoctamin-3

		HNF1A	Hepatocyte nuclear factor 1-alpha
		IER3	Radiation-inducible immediate-early gene IEX-1
		HS3ST2	Heparan sulfate glucosamine 3-O-sulfotransferase 2
		NGEF	Ephexin-1
		IL11	Interleukin-11
		ABCB6	ATP-binding cassette sub-family B member 6
		MTFP1	Mitochondrial fission process protein 1
		SLC7A11	Cystine/glutamate transporter
		ABCC3	Canalicular multispecific organic anion transporter 2
		SLCO2B1	Solute carrier organic anion transporter family member 2B1
		C8orf58	Uncharacterized protein C8orf58
		HK2	Hexokinase-2
		F2RL2	Proteinase-activated receptor 3
		MICALL2	MICAL-like protein 2
		ANGPTL4	Angiopoietin-related protein 4
		MAP2K1	Dual specificity mitogen-activated protein kinase kinase 1
		GTPBP2	GTP binding protein 2
		ISG20	Interferon-stimulated gene 20 kDa protein
		DDIT4	DNA damage-inducible transcript 4 protein
		RIN1	Ras and Rab interactor 1
		FAM57A	Protein FAM57A
		CHRNA9	Neuronal acetylcholine receptor subunit alpha-9
		TNIP1	TNFAIP3-interacting protein 1
		AQP10	Aquaporin-10
		TMEM45A	Transmembrane protein 45A
		PHLDA2	Pleckstrin homology-like domain family A member 2
		P4HB	Protein disulfide-isomerase
		ARHGEF37	Rho guanine nucleotide exchange factor 37
		NXPH4	Neurexophilin-4
		EDARADD	Ectodysplasin-A receptor-associated adapter protein
		ANKRD37	Ankyrin repeat domain-containing protein 37
		ATF3	Cyclic AMP-dependent transcription factor ATF-3
		MYH7	Myosin-7
		DOK3	Docking protein 3
		SERTAD1	SERTA domain-containing protein 1
		AKR1B10	Aldo-keto reductase family 1 member B10
		MAGEC2	Melanoma-associated antigen C2
		MT-ND6	NADH-ubiquinone oxidoreductase chain 6
		BNIP3	BCL2/adenovirus E1B 19 kDa protein-interacting protein 3
		PGAM1	Phosphoglycerate mutase 1
		PGM1	Phosphoglucomutase-1
		GNAS	Guanine nucleotide-binding protein G(s) subunit alpha isoforms XLas
		BEND5	BEN domain-containing protein 5
		IL13RA2	Interleukin-13 receptor subunit alpha-2
		FUT11	Alpha-(1
		SRPX2	Sushi repeat-containing protein SRPX2
		PGK1	Phosphoglycerate kinase 1
		SPAG4	Sperm-associated antigen 4 protein
		COX4I2	Cytochrome c oxidase subunit 4 isoform 2
		PTPRH	Receptor-type tyrosine-protein phosphatase H
		PFKP	ATP-dependent 6-phosphofructokinase
		SRXN1	Sulfiredoxin-1
		PDK1	[Pyruvate dehydrogenase (acetyl-transferring)] kinase isozyme 1
		NDUFA4L2	NADH dehydrogenase [ubiquinone] 1 alpha subcomplex subunit 4-like 2

		SPP1	Osteopontin
		ALDOA	Fructose-bisphosphate aldolase A
		AK4	Adenylate kinase 4
		ODAM	Odontogenic ameloblast-associated protein
		GPR146	Probable G-protein coupled receptor 146
		IRF7	Interferon regulatory factor 7
		ENSG00000164096	Uncharacterized protein C4orf3
		P4HA2	Prolyl 4-hydroxylase subunit alpha-2
		IGF2	Insulin-like growth factor II
		TTC39A	Tetratricopeptide repeat domain containing
		PDZK1	Na(+)/H(+) exchange regulatory cofactor NHE-RF3
		PPP1R13L	RelA-associated inhibitor
		NDRG1	Protein NDRG1
		CT47A11	Cancer/testis antigen family 47
		GBE1	1,4-alpha-glucan-branching enzyme 1
		SLC2A1	Solute carrier family 2
		FAM162A	Protein FAM162A
		SH3D21	SH3 domain containing 21
		LGALS8	Galectin 8
		ADM	ADM
		ENO2	Gamma-enolase
		PFKFB3	6-phosphofructo-2-kinase/fructose-2
		LDHA	Lactate dehydrogenase A
		DDIT3	DNA damage-inducible transcript 3 protein
		GPR56	Adhesion G-protein coupled receptor G1
		CCDC64B	BICD family-like cargo adapter 2
		SLC16A3	Monocarboxylate transporter 4
		HIST1H2BO	Histone H2B type 1-O
		MTHFD1L	Monofunctional C1-tetrahydrofolate synthase
		GREM1	Gremlin-1
		HIST1H2BH	Histone H2B type 1-H
		NUDT18	8-oxo-dGDP phosphatase NUDT18
		HIST1H2BB	Histone H2B type 1-B
